# Goal Setting and Anchoring Effects on Meditation Using a Digital Platform: Large-Scale Digital Field Study

**DOI:** 10.2196/85801

**Published:** 2026-04-09

**Authors:** Michael Bowen, Michael Beam

**Affiliations:** 1 Kent State University, 1800 E Main Street, Kent, OH, 44242, United States, 1 330 672 3000; 2 Spotify (Sweden), Stockholm, Sweden

**Keywords:** goal setting, commitment, precommitment, anchoring, nudging, meditation, mindfulness, app, mobile

## Abstract

**Background:**

Meditation has grown in popularity in recent years, but many people who try meditation often fail to establish a habit. Goal setting has been demonstrated to be an effective technique in behavior change in other health-related contexts but is understudied in the meditation context.

**Objective:**

This study had 2 objectives: (1) to assess the association between goal setting and the number of days people meditated and (2) to evaluate whether anchoring bias in the goal-setting question (via response option order) influences goal selection and subsequent meditation behavior.

**Methods:**

This large-scale quasi-experimental field study included 18,559 Spotify mobile users aged 18 years or older residing in Australia, Canada, New Zealand, the United Kingdom, or the United States who had listened to at least 5 minutes of meditation content from a specified teacher. The in-app experiment consisted of 2 goal-setting test conditions and an active control. In the test conditions, participants selected the number of days they intended to listen to content from the meditation teacher in the next 7 days. The conditions differed only in the order of goal response options (higher goals listed first vs last). The active control rated how much they liked the teacher but did not set a goal. Because responding was optional, selection bias is possible, and the design is quasi-experimental.

**Results:**

The act of setting any goal had a modest positive association with the number of days people meditated in both treatment condition 1 (*β*=.08, 95% CI 0.01-0.16) and treatment condition 2 (*β*=.08, 95% CI 0.002-0.15). People who committed to higher goals were also more likely to meditate more than those who committed to lower goals. Additionally, the distribution of goals between the treatment conditions varied (*χ*^2^_2_=84.24; *P*<.001), and the differences in these distributions subsequently yielded differences in the number of days each group meditated, on average (*t*_2744.1_=−2.34; *P*=.02; Cohen *d*=−0.09). Ultimately, placing the highest goal as the first answer choice yielded higher average active days among those who chose a goal, but many more people opted out of answering the question itself.

**Conclusions:**

Simply offering an optional in-app goal-setting prompt, the intention-to-treat estimate from the experiment, did not change meditation engagement at the population level. However, among users who chose to respond and set a goal, goal selection was associated with a modest increase in active days. Response option order (anchoring) shifted both goal selection and opt-out rates. These findings highlight an uptake-engagement trade-off that is relevant for digital behavior change design.

## Introduction

### Background

Meditation has many demonstrated health benefits [1-3], and in recent years, meditation has been increasing in popularity [4]. Many meditators use meditation apps and streaming services to support their practices [5,6]; however, many people who have tried meditation no longer meditate [6], and retention with digital meditation apps and content, in particular, is low [7-9].

Evidence suggests that greater home meditation practice frequency is associated with better outcomes, highlighting the importance of continued practice [10,11]. Developing a new habit, such as meditation, requires repeated behavior that can develop into automaticity [12-15]. One effective strategy to support this process is planning ahead and setting specific goals, typically expressed in terms of when, where, and how much one intends to engage in the behavior. Goal setting has been shown to increase engagement across a variety of domains, including physical activity, healthy eating, and other health-promoting behaviors [16-19].

Goal setting is an important determinant of goal commitment and pursuit [20], which is a key component of many behavior change theories, both directly in social cognitive theory [21] and through related constructs, such as perceived behavioral control in the theory of planned behavior [22]. For instance, being actively involved in one’s own goal setting can yield positive outcomes [20], and stating a goal publicly can enhance commitment to the goal [23], consequently increasing the belief in one’s ability to achieve the goals. As such, understanding the influence of goal setting in behavior change is important to test key theories of behavioral regulation and habit formation.

Within the context of meditation, action and coping planning strategies and attitudinal commitments have been positively associated with the number of days practicing meditation [24,25]. However, explicit goal setting and goal-intention effects in meditation are understudied, particularly through rigorous techniques. This knowledge gap is especially noteworthy because of the role that goal setting can play in prominent behavior change theories (eg, social cognitive theory, theory of planned behavior). Additionally, goal setting is associated with behavioral outcomes, and practice frequency is crucial for realizing the mental health benefits of meditation, including benefits from using meditation apps and digital content [26-30].

When setting goals, the measurement interface used to elicit the goal is also an important and understudied component of the experience, particularly in the context of meditation. Cognitive psychology has demonstrated the influence of anchoring. For example, the starting points and positions of choices and the subsequent effects these have on people’s judgments and choices [31]. Along these lines, in surveys and survey question design, anchoring biases are a well-known phenomenon that can impact the response distribution [32-34]. In fact, people even rate their success in life differently in surveys when the numeric values on a scale differ [35]. These effects may be explained by dual-process models of judgment, in which people rely on fast, heuristic processing when making quick decisions [31]. In the case of goal commitment within a mobile app, it is plausible that individuals “satisfice” by selecting the first option they come across that seems reasonable, thus making response order a plausible driver of goal commitment. From the perspective of goal-setting theory, anchors that shift potential goals may also change perceived goal difficulty and feasibility, which may influence commitment, self-efficacy, and intention formation [20].

When the objective of asking a question is not explicitly to gather unbiased data for research purposes but rather to encourage people to meditate more, standard survey best practices, such as choice randomization, may be less important than optimizing the goal-setting intervention to outcomes. In a way, these techniques are commonly found in push polls, which are not scientific surveys but rather political persuasion techniques framed as surveys. These kinds of techniques, in which questions have an overt design bias, have been demonstrated to be effective for shifting both memory and opinions [36,37]. These kinds of techniques have also been used in the health behavior goal-setting context. For example, introducing anchoring has demonstrated that people set higher goals when questions are framed as sacrifices (what to forgo) rather than actions (what to undertake) [38]. Consequently, it is important for theorists, applied researchers, and user experience (UX) designers to further understand the effects of anchoring in goal setting, including in the context of meditation. By doing so, these insights can be leveraged for positive impacts on people’s mental health.

### Research Purpose

With these 2 streams of literature in mind, the purpose of this research was twofold. The first and primary objective was to identify the behavioral association between goal setting and engaging with meditation content through a large-scale digital platform. In doing so, we assessed whether people who preemptively set a goal are more likely to meditate than people who did not select a meditation goal. This objective resulted in the following hypothesis:

- H1: People in the treatment conditions who preemptively state a meditation goal will have more days listening to guided meditation teachings during the 7 days following the intervention, on average, compared to the control group.

The second objective was to explore the effects of anchoring on goal intentions, specifically, whether anchoring different goals in the goal-setting question changes the ways in which people set their goals and subsequently how the anchoring effect impacts meditation behaviors. This objective resulted in the following research hypotheses:

- H2: The effects of anchoring preemptively stated meditation goals in the goal-setting question will result in a different distribution of goal intentions.
- H3: Among people who preemptively state a goal, the effects of anchoring different meditation goals in the goal-setting question will result in varying levels of active days with the meditation content during the 7 days following the intervention.

By exploring these hypotheses, this research can support meditation and mental health app product managers in considering whether to implement goal-setting features in their UX and to provide guidance on how to design the feature. Additionally, the present study can help future researchers who study meditation habit formation, particularly with digital health platforms, understand the role of goal setting within the broader context of behavior change.

## Methods

### Sampling and Recruitment

This research was conducted among 18,559 users of the Spotify mobile app. To qualify to participate in the study, individuals needed to have listened to at least 5 minutes of content provided by a popular meditation teacher through the Spotify app during the month of June 2025. The content offered by the meditation teacher consisted of both guided meditations and teachings. Additional qualifications included being aged 18 years or older and residing in the United States, United Kingdom, Canada, Australia, or New Zealand. These countries were chosen because Spotify is available in these countries, the meditation content is in English, and these countries are predominantly English-speaking. Fieldwork was conducted from July 2, 2025, to July 8, 2025. Table 2 in the Results section includes the total sample sizes, country, age, and gender composition of the research sample for each experimental condition. Note that chi-square tests indicated no differences between cells based on country, age, and gender identification.

As the primary experimental intervention, qualified users were assigned to receive 1 of 3 potential one-tap survey questions. Two of the survey questions were treatment conditions, and one was the active control condition. The one-tap survey questions were embedded within a pop-up message in the Spotify mobile app that appeared when a qualified research participant visited the Spotify homepage. Participants could choose to answer the question or dismiss the message without selecting an answer option. Additionally, participants could select a “Prefer not to say” response option if they did not want to select a meditation goal. Upon answering the question, the message was subsequently dismissed automatically, thus ending the intervention.

Because the survey question was optional and, subsequently, goal intention was dependent on a post random assignment event, there was a selection bias in the design when analyzing only people who clicked on the message itself. We detail how this was handled and the implications on the results and interpretation in the Data Analysis Plan section.

### Meditation Content

The meditation content evaluated in this study consisted of a large, publicly available audio catalog produced by a single teacher and distributed as a long-running mindfulness podcast. The catalog includes both (1) guided meditation sessions and (2) didactic “teachings” that provide instruction and reflection intended to support practice. Content primarily reflects mindfulness-based approaches and frequently incorporates self-compassion–oriented instruction (eg, attention to breath/body, present-moment awareness, and working with difficult emotions). Episodes vary in length but tend to be between 20 and 90 minutes. The catalog spans more than a decade and contains over 1500 audio items, often with multiple new items released per week.

### Experimental Design

Each test condition comprised a single survey question. The 2 treatment conditions were variants of the goal-setting question. Specifically, each goal-setting message requested the participant to select the number of days in the next 7 days they intended to listen to guided meditations and teachings offered by the teacher on Spotify. The variants were nearly identical, with only the question response options scale being flipped, which was designed to test the anchoring effect of having the highest vs the lowest number of days response option at the top of the response battery.

Specifically, treatment 1 asked how many days in the next 7 days the user intended to listen to meditation and teachings offered by the teacher on Spotify. Seven days were chosen because goal-setting theory argues that proximal (short-term) goals can facilitate the performance of complex tasks [20], of which meditation could reasonably be considered. The question response options were listed from top to bottom as “0 days,” “1 day,” “2-3 days,” “4 days or more,” and “Prefer not to say.” In treatment 2, the question itself was the same, but the options were reversed, so from top to bottom, the options were listed as “4 days or more,” “2-3 days,” “1 day,” “0 days,” and “Prefer not to say.” Note that selecting “Prefer not to say” was offered as a survey response and was not the same interaction as dismissing the message without selecting any response. However, selecting “Prefer not to say” was not treated as having selected a goal.

 The control condition asked participants how they felt about the meditation and teachings offered by the same meditation teacher on Spotify, with the response options listed from top to bottom as “I love them,” “I like them,” “I have no opinion,” “I dislike them,” and “Prefer not to say.” In using this type of active control, we ensured that the treatment and control conditions had similar interventions, thus equalizing the cognitive effort of responding to a question, controlling for question-behavior effects (where answering any question can influence subsequent behavior), and avoiding inadvertently inducing goal-setting or planning cognitions.

See Table 1 for sample sizes based on participants’ interactions with the goal-setting question.

**Table 1.** Sample sizes based on message interactions.

	Saw the message (all participants), n (%)	Survey responders^a^, n (%)	Selected a goal^b^, n (%)
Control	6012 (100)	2428 (40.4)	2249 (37.4)
Treatment 1	6320 (100)	2065 (32.7)	1542 (24.4)
Treatment 2	6227 (100)	2155 (34.6)	1349 (21.7)

a Participants who did not dismiss the message and chose an answer to the one-tap survey question.

b Participants who did not dismiss the message and did not select “Prefer not to say” as their response.

Following this brief survey-based intervention, we used trace behavioral data from the Spotify app to assess how often users streamed the specific content offered by the respective meditation teacher on the Spotify app.

### Active Days Variable

We define active days as the number of days a participant streamed content from the meditation teacher on Spotify. The postintervention active-days measure (days 0‐7 after the intervention) is the dependent variable for H1 and H3. The preintervention active-days measure (the 30 days preceding the intervention) is also included as an independent covariate. To be considered active on a given day, the participant needed to have streamed content from the teacher for any period of time on the respective day (having pressed play and streamed the content for >0 ms). While this is not necessarily indicative of a meditation session having occurred, it avoids otherwise arbitrary definitions of an active day and is a strong indicator of intent. Summary statistics by cohort are shown in Table 3 in the Results section. In all models, both measures were treated as integer counts.

### Data Analysis Plan

The analysis for this study was conducted using Python. The click-through rate of the survey question (the percentage of people who saw the survey question and subsequently chose a response option, as opposed to dismissing it without responding) for each of treatment 1, treatment 2, and the control was 32.7% (2065/6320), 34.6% (2155/6227), and 40.4% (2428/6012), respectively. A chi-square test indicates that these click-through rates are different (*χ*^2^_3_=85.71; *P*<.001). As a result, there is a selection bias introduced into the experimental design. So, while the overall study is a natural field experiment, the analysis of the effects of goal setting is quasi-experimental and should be considered associations.

To test H1, specifically whether people in the treatment conditions who preemptively state a meditation goal will have more days listening to guided meditation teachings during the 7 days following the intervention, on average, compared to the control, a linear regression model was used. The dependent variable in the model was the number of active days on which the participant listened to content from the meditation teacher through the Spotify app during the 7 days after the intervention. While active days is a bounded and nonnormally distributed variable, linear regression is appropriate because it provides a parsimonious and interpretable approximation for mean differences and has been demonstrated to be valid in these conditions when sample sizes are large [39]. This was coded as an integer, with values ranging from 0 to 8. The range is 0 to 8, not 0 to 7, because the day of the intervention, at any point after the question was answered, is included in the count as day 0. Each treatment condition was inserted separately as an independent variable in the model, while the control condition was held out as the reference category. Note that only people who selected a goal were included in the model. To control for past engagement with the meditation content, the number of active days during the 30 days preceding the treatment that the participant listened to meditation content from the meditation teacher through the Spotify app was included as an independent variable. Age was binned into 5 groups and one-hot encoded as 18 to 24, 25 to 34, 35 to 44, 45 to 54, and aged 55 years or older. The 55 years and older group was held out of the model as the reference category. Gender values included female, male, and other and were also one-hot encoded for modeling. Female was held out as the reference category. Country was also inserted into the model, with the United States held out as the reference category.

H2 explored the role of anchoring on the response distributions of goals chosen. To test this hypothesis, we explored the percentage of users who selected each response option in the survey and applied a chi-square test of independence to specifically assess whether these distributions varied.

To test H3, specifically that the anchoring effect between treatment cells would result in different levels of active days between treatment conditions, a 2-sample Welch *t* test was used to assess differences in postintervention active days.

### Ethical Considerations

The study was reviewed and approved as exempt by the Kent State University Institutional Review Board (IRB) under reference number 2145. The consent process was governed by Spotify’s Terms of Service and Privacy Policy, as the experiment was conducted on the platform with its members. The research team and the IRB carefully reviewed these policies and the ethical conduct of the study, concluding that consent was obtained through the Terms of Service and Privacy Policy. The IRB also determined that this study qualifies as a benign behavioral intervention. Personally identifiable information about Spotify users was not accessible to the researchers and was safeguarded through encryption. It can only be accessed through a review and approval process by the Spotify Data Protection Office. No compensation for participation was provided.

## Results

Table 2 provides the demographic composition of the sample. As previously noted, chi-square tests indicated no differences between cells based on country, age, and gender identification.

Table 3 provides the descriptive statistics for the number of active days before and after the treatment based on the varying levels of engagement with the message.

**Table 2.** Demographic composition of experimental conditions.

	Control (N=6012), n (%)	Treatment 1 (N=6320), n (%)	Treatment 2 (N=6227), n (%)
Country			
Australia	631 (10.5)	653 (10.3)	632 (10.1)
Canada	756 (12.6)	767 (12.1)	787 (12.6)
United Kingdom	819 (13.6)	897 (14.2)	917 (14.7)
United States	3684 (61.3)	3867 (61.2)	3733 (59.9)
New Zealand	122 (2)	136 (2.2)	158 (2.5)
Age (years)			
18-24	234 (3.9)	233 (3.7)	244 (3.9)
25-34	1520 (25.3)	1570 (24.8)	1520 (24.4)
35-44	1898 (31.6)	1975 (31.3)	1935 (31.1)
45-54	1223 (20.3)	1272 (20.1)	1275 (20.5)
≥55	1137 (18.9)	1270 (20.1)	1253 (20.1)
Gender identification^a^			
Female	4453 (74.1)	4601 (72.8)	4605 (74)
Male	1257 (20.9)	1394 (22.1)	1344 (21.6)
Other/prefer not to say	59 (1)	72 (1.1)	53 (1)

a Gender data is collected at the time of Spotify registration. It is provided to Spotify optionally and is not available for all participants.

**Table 3.** Summary statistics for “active days.”

Time window	Role in models	Cohort	Mean (SD)
7 days postintervention	Dependent (H1, H3)	All participants	0.95 (1.5)
7 days postintervention	Dependent (H1, H3)	Survey responders^a^	1.11 (1.62)
7 days postintervention	Dependent (H1, H3)	Selected a goal^b^	1.18 (1.66)
30 days preintervention	Independent covariate	All participants	3.93 (4.31)
30 days preintervention	Independent covariate	Survey responders^a^	4.27 (4.52)
30 days preintervention	Independent covariate	Selected a goal^b^	4.49 (4.66)

a Participants who did not dismiss the message and chose an answer to the one-tap survey question.

b Participants who did not dismiss the message and did not select “Prefer not to say” as their response.

Before testing the specific hypotheses listed previously, we first explored whether simple exposure to the commitment message (ie, participants saw the message) affected the likelihood of participants in the control versus treatment conditions to have more active days following the intervention. This could be considered the intention-to-treat effect from the messages and the results of the true field experiment component of the research. A Welch *t* test showed that the overall mean number of active days between the control condition (mean 0.94, SD 1.48 days) and the treatment conditions combined (mean 0.96, SD 1.51 days) was not different (*t*_12,045.6_=−0.76; *P*=.45).

In the linear regression used to test H1, participants in both treatment condition 1 (*β*=.08, 95% CI .01-.16) and treatment condition 2 (*β*=.08, 95% CI .002-.15) had more average active days with the meditation content compared to the control group. The model controlled for preintervention activity levels, as well as the specific goals and answer choices participants selected, among others, indicating that simply selecting a goal is associated with increased active days. Therefore, we can reject the null hypothesis for H1.

Additionally, people who set higher goals for themselves tended to have more active days on average. The adjusted *R*² for this model was 0.396. See Table 4 for more details on the linear regression model of days active after the intervention.

**Table 4.** Coefficient, upper and lower bound CI (95%), and *P* value from linear regression of posttreatment days active.

Variable	Coefficient (95% CI)	*P* value
Constant	0.19 (0.08 to 0.30)	<.001
Preexposure days active	0.21 (0.20 to 0.22)	<.001
Experimental condition		
Control	—^a^	—
Treatment 1	0.08 (0.01 to 0.16)	.03
Treatment 2	0.08 (0.002 to 0.15)	.05
Country		
Australia	0.09 (−0.04 to 0.21)	.17
Canada	0.09 (−0.03 to 0.21)	.13
United Kingdom	0.05 (−0.06 to 0.16)	.34
United States	—	—
New Zealand	0.28 (0.06 to 0.50)	.01
Gender identification		
Female	—	—
Male	0.08 (0 to 0.17)	.06
Age (y)		
18‐24	−0.36 (−0.54 to −0.19)	<.001
25‐34	−0.22 (−0.33 to −0.11)	<.001
35‐44	−0.22 (−0.33 to −0.11)	<.001
45‐54	−0.07 (−0.19 to 0.04)	.22
55 or older	—	—
Goal intention survey response		
0 days	−0.21 (−0.29 to −0.14)	<.001
1 day	−0.05 (−0.12 to 0.03)	.23
2‐3 days	0.08 (0 to 0.16)	.04
4 days or more	0.34 (0.23 to 0.44)	<.001
Prefer not to say	—	—
Control survey response		
I dislike them	0.01 (−0.34 to 0.37)	.94
I have no opinion	−0.10 (−0.29 to 0.09)	.30
I like them	−0.02 (−0.15 to 0.10)	.70
I love them	0.14 (0.05 to 0.23)	.003
Prefer not to say	—	—

a Not applicable.

**Figure 1.**
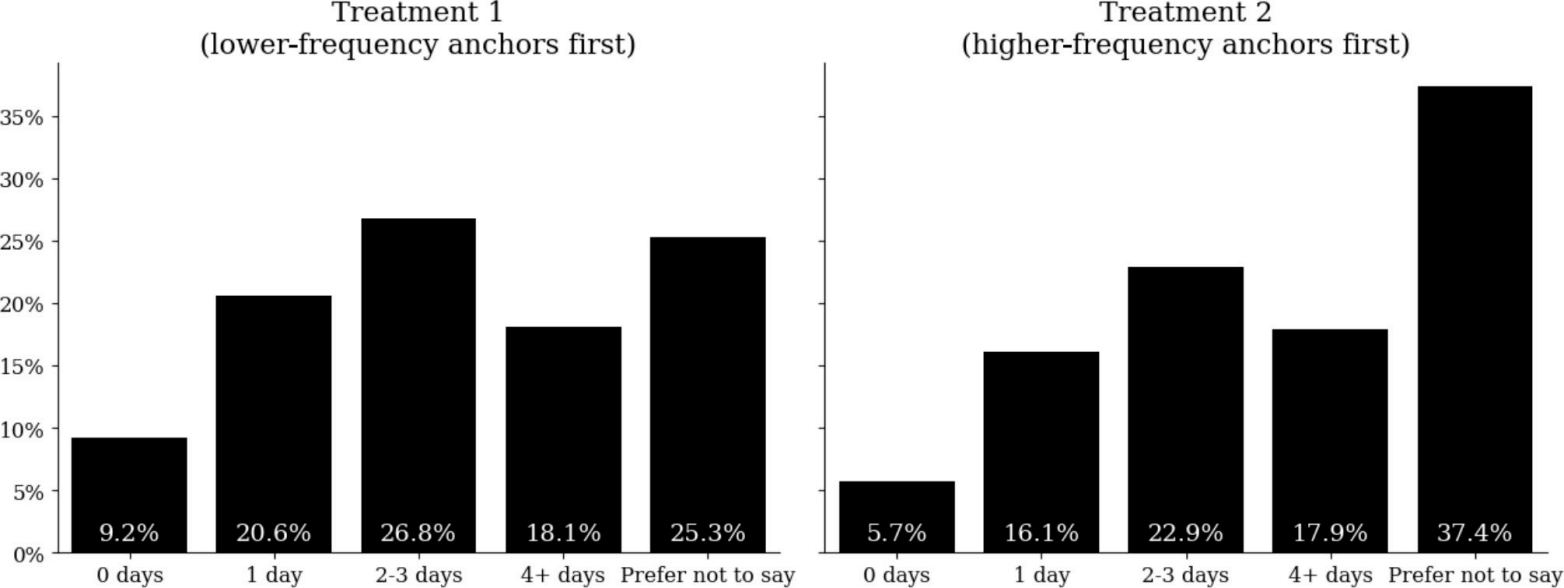
Distribution of goal intention one-tap survey responses based on treatment condition (percentage of users who chose their respective meditation goal by treatment condition).

To test whether the ordering of the goal response options created an anchoring effect that influenced the distribution of responses in H2, a chi-square test of independence was used. Figure 1 includes the distribution of responses from the survey within the 2 test conditions. The test yielded (*χ*^2^_2_=84.24; *P*<.001), indicating significant differences in the distribution of responses across treatment conditions. When the “Prefer not to say” response is removed from the distribution and the chi-square statistic recalculated, the result yielded (*χ*^2^_2_=13.03; *P*=.005), indicating that the anchoring effect persists. As a result, we can reject the null hypothesis for H2.

In H3, the Welch *t* test showed that the mean number of active days differed between treatment 1 (mean 1.15, SD 1.62 days) and treatment 2 (mean 1.3, SD 1.78 days; *t*_2744.1_=−2.34; *P*=.02). A Cohen *d* of −0.09 indicates a modest overall effect of the differences in days active between treatment conditions. As a result, we can reject the null hypothesis for H3. It is important to note, however, that when we included people who did not select a goal (ie, instead they selected “Prefer not to say”), the effect is not significant (*t*_4217.7_=−0.68; *P*=.50).

Table 5 provides a summary of the hypotheses, test results, and decisions. Additionally, a CONSORT (Consolidated Standards of Reporting Trials) diagram of the full experimental setup is available in Multimedia Appendix 1.

**Table 5.** Summary of hypotheses, test results, and decisions.

Hypothesis	Hypothesis (abridged)	Test (*P* value)	Decision
H1	People who preemptively state a meditation goal will have more days listening to guided meditation teachings during the 7 days following the intervention, on average, compared to the control.	Participants who stated a meditation goal were more likely to engage with the meditation content in both Treatment 1 (*β*=.08, 95% CI 0.01-0.16) and Treatment 2 (*β*=.08, 95% CI 0.002-0.15), compared to the control.	Reject null hypothesis
H2	The effects of anchoring different meditation goals in the treatment conditions will result in a different distribution of goal intentions.	*χ*^2^ (2) = 84.24 (*P*<.001)	Reject null hypothesis
H3	The effects of anchoring different meditation goals in the goal-setting question will result in varying levels of active days with the meditation content during the 7 days following the intervention.	*t* (2744.1) = −2.34 (*P*=.02)	Reject null hypothesis

## Discussion

### Principal Results

This study demonstrated that setting short-term goals for guided meditation and teachings through a mobile app was associated with engaging with the meditation content on more days. Specifically, people who preemptively set themselves any goal were more likely to meditate on more days than people in the control condition. Additionally, people who chose higher goals were more likely to engage with meditation content more often, even after controlling for past engagement with the meditation content, as well as age, gender, and country of residence. Conversely, people who selected zero as a goal meditated less often. While the differences were modest, these uptake-conditional results suggest that among participants who elected to respond, goal selection was associated with slightly more active days. The intention-to-treat analysis indicated that simply offering the optional goal-setting prompt (thus including people who chose to ignore the message) did not influence subsequent meditation behavior.

This finding adds to the body of literature that goal setting may be useful in health behavior change in the context of meditation, similar to other health behavior contexts such as physical activity and nutrition [16-19]. Like Epton et al [16] found in their meta-analysis of the effects of goal setting across a range of behaviors, the effect of goal setting from this research was found to be small and should subsequently be considered just one, perhaps small, component of meditation habit formation. However, while the overall effects may be small, at scale, these kinds of cost-effective nudges can be worthwhile interventions [40]. For meditation content providers and program managers, these findings provide evidence that goal-setting interventions may be useful, if modest, tools to increase engagement with meditation content. Ultimately, for anyone who has a desire to develop a meditation habit, this study provides compelling data that goal setting may be worthwhile in building and maintaining a meditation habit.

This study also provides clear evidence of an anchoring effect based on how the goal-intention question is structured. Specifically, when higher goal response options are anchored at the top of the response battery, the response distributions change substantively. Most notably, many more people selected “Prefer not to say” when the “4+ days per week” option was anchored at the top of the response battery compared to the bottom (37.4% vs 25.3%). This is likely explained by dual-process models of judgment, in which individuals rely on quick, heuristic processing when making low-effort decisions [31]. Within this study environment, individuals likely chose the first plausible goal. Seeing the most ambitious goal at the top may have felt unrealistic or hindered self-efficacy for some people, both of which are important components of goal setting and goal accomplishment [20]. Additionally, people who were in the Treatment 2 condition and selected a meditation goal (ie, did not select “Prefer not to say”) subsequently engaged with meditation slightly more than people in Treatment 1 who selected a goal, suggesting that the anchoring effect may not have only affected the response distribution but also had a modest effect on people’s propensity to meditate.

These findings indicate that there are trade-offs to anchoring. While it is best practice in survey research to randomize response options or randomly flip ordered scales in survey design [32-34], practitioners and UX designers should consider the outcomes they want to achieve when choosing anchors and whether to flip scales. Push polls are ethically fraught cautionary tales of the impact of asking biased questions [36,37]. However, in the context of health behavior, this technique may lead people to set more ambitious goals [38].

Overall, this study provides theoretical insights about the role of goal setting in meditation habit development and maintenance, adding to the body of literature about goal setting in different contexts. It also offers new insights into the effects of anchoring on goal setting within the meditation context. Finally, the research provides UX designers and product managers practical guidance on the potential impact of goal setting and anchoring on user engagement.

### Limitations

There are several limitations to this research that should be considered. First, as described previously, because people could opt out of participating in the one-tap survey, there is a self-selection bias that makes this research a quasi-experimental design and not a full field experiment. Consequently, we cannot confidently conclude that we have established a causal association between goal setting and incremental meditation. While this is an important limitation in drawing conclusions, the nature of the research itself reflects real-world environments and digital platform experiences.

Second, this study was conducted on a single digital platform (Spotify) and includes only a single meditation teacher, which limits the broader generalizability of the findings. Spotify may be unique in that it primarily offers music, podcast, and audiobook content and is not broadly considered a digital mental health services provider. As such, goal-setting exercises on platforms that specialize in digital mental health services may differ. Additionally, the research includes only log behavioral data and does not include important context, such as motivations for meditation and meditation experience. Expanding this research to additional platforms and teachers, to more segments of the meditation population (eg, more experienced meditators who already have strong self-efficacy vs less experienced meditators who need structure), and with additional context (eg, mapping motivations to goal-setting outcomes) could be considered by future researchers.

Additionally, while the single-tap question is a strength of the study because it optimizes response rates and limits nonresponse bias, a single question served as a pop-up has formatting constraints. Consequently, we could not offer more detailed response options and could not probe more deeply into participants’ relationship with meditation. Data of this kind may have been helpful to add more context to the insights offered from the study.

### Future Directions

The results from this study open up additional avenues for exploration for future researchers. First, future studies may strive to make the research a fully experimental design by making the goal-setting question itself mandatory. This would allow researchers to better understand the full causal effects of goal setting within the meditation technology context. Additionally, as described, it would also be useful to study this phenomenon on other apps and in other contexts, which would allow researchers to generalize the effects of goal setting on meditation more broadly.

This research also looked only at the average effect of the treatment across experimental conditions. Future researchers might further explore how goal setting impacts different cohorts of meditators, such as lighter versus heavier meditators or people with a burgeoning versus an established practice. Doing so would allow theorists to better understand the effects of goal setting throughout the stages of meditation habit formation and for UX designers and product managers to strategically target their goal-setting user growth initiatives.

### Conclusion

This research provides insights about the role of goal setting in the meditation context when applied to digital health platforms. Ultimately, goal setting may be a useful and cost-effective tool when applied on a large scale to encourage people to engage with meditation more frequently on digital platforms, although the overall impact may be modest. However, anchoring effects play a significant role in people’s willingness to set meditation goals, the goals they set for themselves, and even their incremental meditation engagement. Researchers who study the theoretical aspects of meditation habit formation can use this research to consider the role of goal setting in their theories and models. Clinical practitioners and UX designers should consider explicit goal setting when encouraging people to practice meditation but think carefully about the effects of anchoring in their designs.

## Supplementary material

10.2196/85801 Multimedia Appendix 1 CONSORT diagram of experimental design and outcomes.

## Abbreviations

CONSORT: Consolidated Standards of Reporting Trials
IRB: institutional review board
UX: user experience
