# Supplementary figures and images for: Goal Setting and Anchoring Effects on Meditation Using a Digital Platform: Large-Scale Digital Field Study
Source: J Med Internet Res. 2026 Apr 9;28:e85801. doi: 10.2196/85801 (PMC13065232; doi:10.2196/85801)

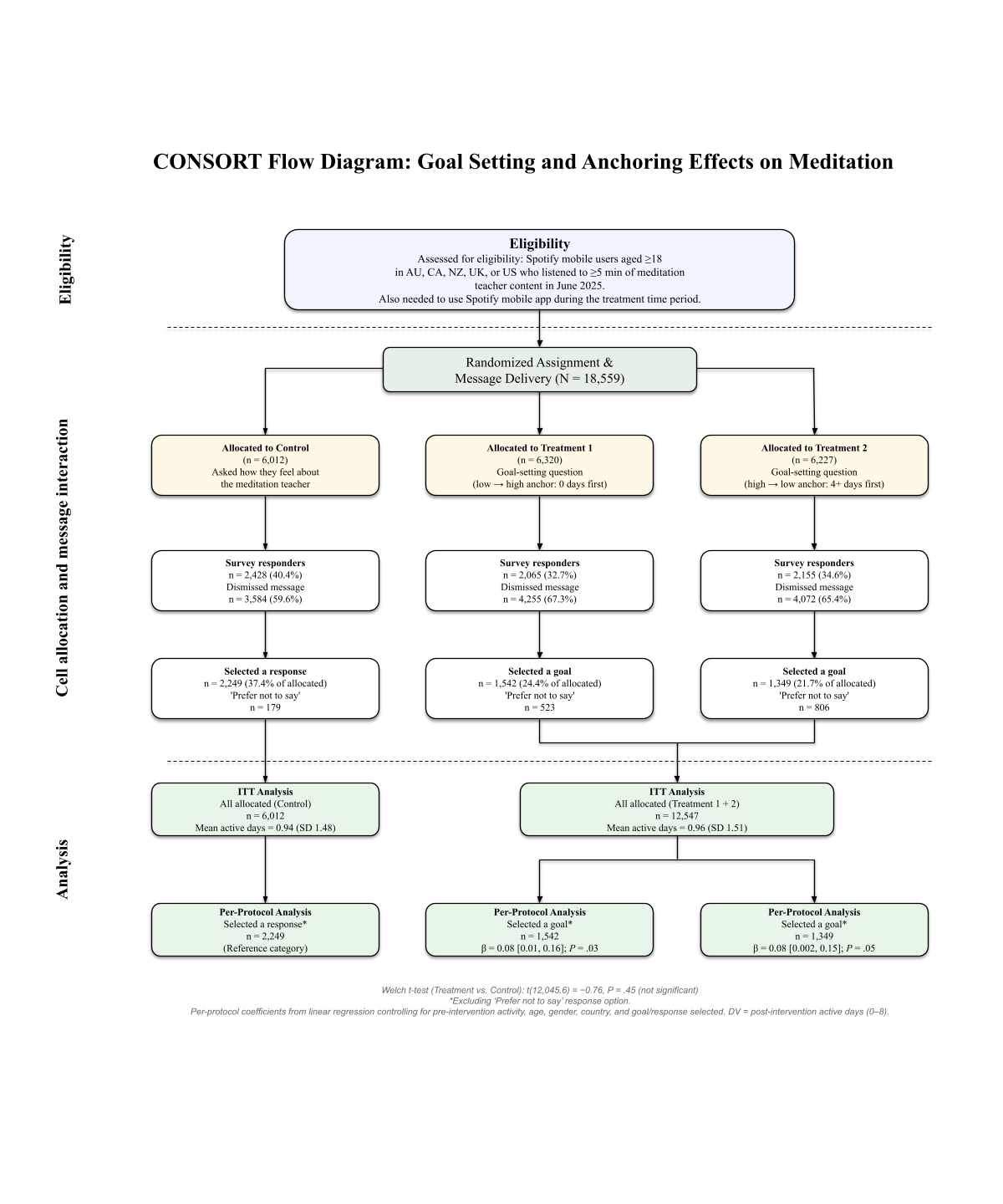

Supplement: Multimedia Appendix 1 [file jmir-v28-e85801-s001.png]
